# The influence of members’ attachment style on group cohesion in substance use therapy groups

**DOI:** 10.1007/s44202-022-00022-w

**Published:** 2022-01-24

**Authors:** Anissa Naeli, Melina Skentzos, Anastasia Hronis

**Affiliations:** Graduate School of Health, University of Technology Sydney, Sydney, Australia

## Abstract

Group cohesion is an important factor in group therapy as it influences treatment outcomes and group processes. One’s attachment style has been found to impact experiences of group cohesion, however research into this relationship in substance use disorder (SUD) group treatment is lacking. This is of particular concern as insecure attachment presentations are more prevalent in this clinical population and group therapy is often treatment of choice. The current study sought to determine whether attachment style significantly predicted group cohesion. It also provided a qualitative exploration of factors that promote group cohesion in SUD group treatment. Participants (*N* = 38) attending a SUD therapy program completed self-report measures. Attachment avoidance was a significant negative predictor of positive bonding and positive working, and a significant positive predictor of negative relationships. Attachment anxiety was a significant negative predictor of negative relationships, however, unexpectedly did not significantly predict positive bonding or positive working. The current findings highlight the importance of group therapists tailoring group treatment to meet the attachment needs of individuals to promote a group therapy environment conducive to recovery.

## Introduction

### Substance use in Australia

The use of alcohol and other drugs (AOD) in Australia has a significant social, economic and health impact at the individual, family and community level [1]. In 2019, 7.5% of Australians aged 14 and over were likely to meet criteria for alcohol dependence, while 43% reported having used an illicit drug during their lifetime [1]. Clinically, a substance use disorder (SUD) is a cluster of cognitive, behavioural, and physiological symptoms indicating that the individual continues to use the substance despite significant substance-related problems. Diagnosis is based on features of hazardous substance use, impaired control over use, tolerance, withdrawal, and impact on physical, psychological and social functioning [2]. Given the prevalence and impact of AOD use in Australia, research into effective SUD treatments is necessary for clinicians, patients, and the systems within which therapies are delivered.

### SUD group treatment

Group therapy for SUDs is often a treatment of choice. Meta-analyses have found little to no difference in the effectiveness and efficacy of group compared to individual SUD therapy [3, 4], however randomised controlled trials have found group treatment to be more cost- and time-effective [5, 6]. Furthermore, the group format provides opportunities conducive to recovery that are not present in individual therapy. These include the ability to witness the recovery of others, reduce social isolation, and facilitate peer support and mutual learning between members [7]. Group therapy also helps to alter faulty self-concepts as members receive feedback from one another, and fosters a culture of hope and recovery [7].

The integration and generalisability of findings across the SUD group therapy literature is challenging, due to varying factors specific to the format of the treatment. There are variations in treatment modality (e.g. cognitive behavioural therapy [CBT], interpersonal processing groups), treatment setting (e.g. residential, outpatient) and participant specific factors (e.g. mixed or specific gender, mandated or voluntary admission) [8, 9]. This creates difficulties in comparing research outcomes, highlighting a need for future research to explore how individual client characteristics and underlying group mechanisms influence processes and treatment outcomes [9]. This would allow for easier synthesis and application of research to clinical practice, improving clinicians’ capacity to provide evidence-based group treatment tailored to the individual patient.

### Group cohesion

Yalom & Leczsz [10] proposed 11 therapeutic factors integral and unique to the experience and outcomes of group therapy. Of these factors, group cohesion has been considered the most important [10, 11]. It was originally defined by Yalom & Leczsz [10] as the result of all the forces acting on all the members such that they remain in the group, or, more simply, the attractiveness of a group for its members. Meta-analyses have found it to be an important predictor of treatment outcomes across different clinical populations [12, 13]. It has also been associated with other important group processes, such as increased self-disclosure and ability to tolerate conflict within groups [14, 15]. In SUD populations, there have been mixed findings on group cohesion. Some research has found a significant relationship between group cohesion (or group relationships more broadly), and group processes and outcomes [16–21]. Other research has found no significant association [22, 23], or found it to be related to other outcome variables, such as psychological distress, however not reductions in substance use [24]. Once again, there is difficulty synthesising research due to the various factors present in group therapy. Given the theoretical and empirical support for the use of group treatment with SUD clinical populations, and strong research highlighting the importance of cohesion in successful group treatment, further research into group cohesion in SUD treatment groups is worthwhile.

Another challenge presented by the existing literature is the inconsistency in definitions of group cohesion. This impacts the ability to synthesise and develop research in this area. Burlingame et al. [12] outlined eight common self-report measures used to assess cohesion, with each varying in their conceptual and operational definitions of the term. In an attempt to consolidate commonly used measures and definitions of group relationship (i.e. group -cohesion, -climate, -alliance and -empathy), Johnson et al. [25] developed a three-factor model of group relationship. They proposed group relationship is comprised of three factors, (a) *positive bonding (PB)*, the sense of belonging or attraction a member has to the group, its members; and its leader(s), (b) *positive working (PW)*, the ability of the group to agree upon and work toward treatment goals in an effective manner; and (c) *negative relationship (NR)*, the lack of trust, genuineness, and understanding, as well as friction and distance that may exist between the group, its members and/or its leaders [25]. The current study uses this model to define group cohesion.

### Attachment theory

Group cohesion is an important predictor of positive treatment outcomes and processes, thus a deeper understanding of how to cultivate it is vital. Tucker et al. [26] examined predictors of group cohesion and found that individual client characteristics, such as gender and attachment style, accounted for 80–97% of change in cohesion. Attachment theory [27, 28] refers to one’s tendency to seek proximity to significant others in times of need, shaped by their early experiences with caregivers. The current study uses a two-dimensional model of attachment style [29]. The first is *attachment anxiety*, an individual’s excessive need for reassurance, fear of rejection, and a desire to merge with relationship partners, and the other is *attachment avoidance*, the extent to which an individual avoids intimacy and is distrusting of others [30]. High levels of both indicate a more insecure attachment presentation, while lower levels indicate a more secure attachment. Empirical research has found that attachment style influences one’s cognitions, emotions, and behaviours in a group setting, and how they relate to other members, leaders and the group as a whole [31–34]. It has also been found to moderate the relationship between group cohesion and treatment outcomes [35].

### Influence of attachment style on group cohesion

The impact of attachment style on group cohesion appears to differ between attachment avoidance and attachment anxiety. Research has found individuals with stronger attachment avoidance report lower cohesion and weaker group relationships in both clinical and non-clinical populations [26, 31, 36–39]. The impact of attachment anxiety, however, is less clear as some research has found individuals with stronger attachment anxiety report better group cohesion and more positive group relationships [36, 37], while other research has proposed these individuals assess group interactions as threatening and this reduces their ability to work effectively in a group [8]. Further research has found attachment anxiety is not significantly related to initial group cohesion, however predicts growth of cohesion in early stages of group [26]. The attachment style of one group member has also been found to impact other members’ perceptions of the rest of the group [36, 38], and heterogeneity of attachment style within a group has also been associated with stronger PB and lower NR [40]. While the existing empirical findings are mixed, research highlights that attachment style plays a clear role in cohesion within the group therapy context.

Mikulincer and Shaver [41, 42] provide a theoretical understanding of this role, proposing that attachment anxiety and avoidance reflect an individual’s internal working models, which are activated in group therapy settings and influence how they approach and perceive interpersonal interactions. They propose members with stronger attachment anxiety have an internalised positive view of others, combined with a negative view of self, whereas those with stronger attachment avoidance have internalised a negative view of others combined with a positive view of self [41]. They also suggest group cohesion may protect individuals against their insecure working models, as it enhances the likeliness of forming a secure attachment to the group [41]. Leszcz [43] argues this allows the group to serve as a secure base for the individual to “venture, take risks, and explore, knowing they can return to a safe, responsive, and accepting environment” [p. 281]. A clear understanding of the impact of attachment on group cohesion in SUD therapy groups is important, as insecure attachment presentations are more common in SUD clinical populations, having been found to be a risk factor for the presence of AOD related concerns [44–47].

### Current study

Group cohesion has been shown to be an influential factor in group treatment, impacting other important group processes and treatment outcomes. Given that attachment style is a strong predictor of group cohesion [26], and that insecure attachment presentations are prevalent in the SUD clinical population [44–47], an understanding of how members’ attachment style influences group cohesion in SUD group treatment is important. To the author’s best knowledge, there is currently no research exploring this relationship. This study aims to examine whether group members’ levels of attachment avoidance and anxiety predict their experiences of group cohesion in a SUD therapy group, in the domains of PW, PB and NR. Based on existing empirical and theoretical research, the authors hypothesise that stronger attachment avoidance will predict lower PB and PW, and higher NR, while stronger attachment anxiety will predict higher PB and PW, and lower NR. The study also aimed to provide a qualitative exploration of the factors that group members identified as promoting successful group cohesion through qualitative responses.

## Method

### Participants

Participants were individuals attending an outpatient day program for addiction treatment at a private mental health hospital in Sydney. A total of 38 people participated in the study over a period of three months. Of the sample, 44.74% of participants identified as female (*n* = 17), 52.63% identified as male (*n* = 20) and 2.6% preferred not to disclose their gender (*n* = 1). Participants were aged 19 to 65 (*M* = 42.16, *SD* = 13.08), were fluent in English and had a formal diagnosis of a SUD as diagnosed by a psychiatrist. Participants’ demographics are listed in Table 1.

**Table 1:** Participant demographics

Ethnicity	%	*n*
Australian	36.84	14
Mixed Ethnicity	21.05	8
European	15.79	6
Middle Eastern	13.16	5
South American	5.26	2
Asian	2.63	1
Indian	2.63	1
New Zealander	2.63	1
Relationship status		
Married	31.58	12
Single	26.32	10
In a relationship	21.05	8
Separated	10.50	4
Divorced	5.26	2
Widowed	5.26	2
Education		
Year 10	2.63	1
High School	13.16	5
TAFE/College	23.68	9
Bachelor’s Degree	34.21	13
Master’s Degree	13.16	5
Doctorate	2.63	1
No response	10.53	4
Gross individual income		
$0-$20 000	2.63%	1
$20 000-$34 999	2.63%	1
$35 000-$49 999	15.79%	6
$50 000-$74 999	28.95%	11
$75 000-$99 000	23.68%	9
$100 000 +	26.32%	10

% = Percentage of sample (*N* = 38), *n* = number of participants who reported

### Materials

#### Participant demographic information

Participants responded to questions about their gender, age, ethnicity, relationship status, income, education, particular substance(s) of addiction, number of group sessions previously attended and group size for the day.

#### *Kessler-6 (K-6)* [48]

A 6-item self-report measure assessing psychological distress. A total score is calculated by totalling response scores. Total scores can range from 6 to 30, with higher scores indicating more psychological distress. The K-6 is reported to have good test–retest reliability and internal consistency [49].

#### Attachment style questionnaire-short form (ASQ-SF) [50]

A 40-item self-report measure assessing attachment style on two domains, attachment anxiety and attachment avoidance. Total scores for both subscales are produced by calculating the average of the responses to the items assigned to that subscale. Total scores for each subscale can range from 1 to 6, with higher scores on either scale indicating more insecure attachment in individuals. The ASQ-SF is based on the Attachment Style Questionnaire (ASQ) [51], which has been found to have good reliability and validity [52]. Karantzas et al. [30] also reported good reliability and validity for the ASQ-SF.

#### *Group questionnaire* (GQ) [53]

A 30-item self-report measure assessing the quality of therapeutic relationship in a group across three domains, PB, PW, and NR. Scores for each subscale are produced by summing the responses to the items assigned to that subscale. PB scores range from 13 to 91 with higher scores indicating the participant perceives a strong alliance with the group-as-a-whole, leader and other members. PW scores range from 8 to 56 with higher scores indicating the participant experiences the group as meeting their expectations about the changes they seek and as being able to work together on mutually agreed goals. NR scores range from 9 to 63 with higher scores indicating the participant may be experiencing an alliance rupture with the leader, empathic failure from other members or group conflict. The GQ is reported to have good internal consistency and construct validity [54].

#### Qualitative questions on group cohesion

Participants were provided with a description of group cohesion and asked to respond to two qualitative questions, “What do you think leads to better group cohesion?” and “Why is it important for you to experience good group cohesion?”.

### Procedure

This study received ethical approval by the Human Research Ethics Committee (ETH21-5765). Individuals who attended the outpatient day program were recruited on a voluntary basis, and provided consent to participate as well as for results to be published. Participants were invited to anonymously complete paper-based self-report questionnaires and informed that there would be no consequences to their treatment if they participated or declined. Participants were only able to participate once in the study during a single group session. On completion, questionnaires were collected by group facilitators and provided to researchers for analysis.

### Statistical analysis

Three separate multiple linear regression analyses were conducted to determine whether participants’ self-reported attachment style on the domains of avoidance and anxiety significantly predicted (a) PB, (b) PW and (c) NR. Thematic analyses were conducted on qualitative data. Participants’ responses were coded by the main author and cross-checked by the second author.

The authors also intended to run moderation analyses to explore whether client and group characteristics influenced the relationship between attachment style and group cohesion. This could not be conducted as the intended sample size of 100 was not achieved due to the impact of COVID-19. A sample size of 100 would yield a power of 0.8 to detect a beta coefficient of 0.27 (in units of SD of the independent and dependent variable). A sample size of 38 participants would yield a power of 0.8 to detect a beta coefficient of 0.42.

## Results

### Descriptive statistics

Participants reported on their substance(s) of addiction. See Table 2. 68.42% of participants reported one substance of addiction (*n* = 26), 18.42% reported two substances of addiction (*n* = 7), and 13.16% reported three or more substances of addiction (*n* = 5).

**Table 2:** Participants’ reported substances of addiction

Substance	*%*	*n*
Alcohol	97.37	37
Marijuana	18.42	7
Cocaine	10.53	4
Gambling	7.89	3
Benzodiazepines	2.63	1
Methamphetamines	2.63	1
Prescription medication (did not specify drug type)	2.63	1
Pain medication (did not specify drug type)	2.63	1

% = Percentage of sample (*N* = 38), *n* = number of participants who reported the substance of addiction

Participants’ scores of psychological distress on the K6 ranged from 8 to 28 (*M* = 18.26, *SD* = 5.11). Group size on day of study participation ranged from 6 to 14 participants (*M* = 9.18, *SD* = 2.31). The number of group sessions participants had previously attended were reported as 1–10 sessions (47.37%, *n* = 18), 11–20 sessions (26.32%, *n* = 10), 21–30 sessions (7.89%, *n* = 3), 31–40 sessions (2.63%, *n* = 1), 41 + sessions (13.16, *n* = 5), and 2.63% of participants did not respond (*n* = 1). As mentioned, the authors intended to use this data to conduct moderation analyses, however as this could not be conducted, only descriptive statistics have been reported.

Participants attachment avoidance scores ranged from 1.44 to 5.06 (*M* = 3.32, *SD* = 1.01), and attachment anxiety scores ranged from 1.15 to 5.85 (*M* = 3.43, *SD* = 1.00). One participant did not provide a response for question 19 of the ASQ. The single missing item was imputed by averaging the participants’ responses on the attachment anxiety subscale, to which the item pertained. Scores on PB ranged from 69.00 to 91.00 (*M* = 84.11, *SD* = 7.30), scores on PW ranged from 32.00 to 56.00 (*M* = 47.84, *SD* = 7.25), and scores on NRs ranged from 9.00 to 27.00 (*M* = 14.24, *SD* = 5.18).

### Data screening

Assumptions for the three multiple linear regression analyses were checked after the models had been fitted. Partial linear regression scatterplots fit with a LOESS line and scatterplots of studentized residuals against the predicted values indicated linearity between dependent and independent variables. There was no evidence of homoscedasticity from scatterplots of studentized residuals versus unstandardized predicted values. The variance inflation factors were all below 0.1 indicating multicollinearity. The assumptions that residuals were approximately normal was found in Q-Q plots. For analyses 1 and 2, there were no studentized deleted residuals greater than ± 3 standard deviations, no leverage values greater than 0.2 and values for Cook’s distance were above 1, indicating no outliers. For analysis 3, a high studentized deleted residual greater than ± 3 standard deviations was found, indicating a possible outlier. The decision was made to retain this data, as the values were within proper limits, the leverage value was less than 0.2, the Cook’s distance was above 1 and the dfbeta was within appropriate range.

## Main analysis

### Relationship between attachment and PB

A multiple regression analysis was run to determine whether attachment style was a significant predictor of PB within the group. The multiple regression model showed that attachment style significant predicted PB, *F*(2, 35) = 7.59, *p* = 0.002. Attachment avoidance was a significant negative predictor of PB (*p* < 0.001), while attachment anxiety was not a significant predictor (*p* = 0.289). Regression coefficients and standard errors can be found in Table 3.

**Table 3:** Multiple regression results for PB

Positive Bonding	*B*	95% CI for *B*		*SE B*	*R*^*2*^	*R*^*2*^_adjusted_	*p*
		*LL*	*UL*				
Model					0.30	0.26	0.002
Constant	94.66	86.42	102.90	4.06			< 0.001
Attachment avoidance	− 4.58	− 7.09	− 2.07	1.24			< 0.001
Attachment anxiety	1.36	− 1.18	3.90	1.25			0.286

Model = “Enter” method in SPSS statistics; *B* = unstandardized regression coefficient, CI = confidence interval; *LL* = lower limit, *UL* = upper limit, *SE B* = standard error of the coefficient; β = standardized coefficient; *R*^*2*^ = coefficient of determination; *R*^*2*^_adjusted_ = adjusted *R*^*2*^*; p* = probability value

#### Relationship between attachment and PW

A multiple regression analysis was run to determine whether attachment style was a significant predictor of PW within the group. The multiple regression model showed that attachment style significantly predicted PW, *F*(2, 35) = 6.67, *p* = 0.004. Attachment avoidance was a significant negative predictor of PW (*p* < 0.001), while attachment anxiety was not a significant predictor (*p* = 0.099). Regression coefficients and standard errors can be found in Table 4.

**Table 4:** Multiple regression results for PW

Positive Working	*B*	95% CI for *B*		*SE B*	*R*^*2*^	*R*^*2*^_adjusted_	*p*
		*LL*	*UL*				
Model					0.28	0.24	0.004
Constant	55.56	47.21	63.90	4.11			< 0.001
Attachment avoidance	− 4.55	− 7.09	− 2.00	1.25			< 0.001
Attachment anxiety	2.15	− 0.42	4.72	1.27			0.099

Model = “Enter” method in SPSS statistics; *B* = unstandardized regression coefficient, CI = confidence interval; *LL* = lower limit, *UL* = upper limit, *SE B* = standard error of the coefficient; β = standardized coefficient; *R*^*2*^ = coefficient of determination; *R*^*2*^_adjusted_ = adjusted *R*^*2*^*; p* = probability value

#### Relationship between attachment and NRs

A multiple regression analysis was run to determine whether attachment style was a significant predictor of NRs within the group. The multiple regression model showed that attachment style significantly predicted NRs, *F*(2, 35) = 11.12, *p* < 0.001. Attachment avoidance was a significant positive predictor of NRs (*p* < 0.001) and attachment anxiety was a significant negative predictor (*p* = 0.017*)*. Regression coefficients and standard errors can be found in Table 5.

**Table 5:** Multiple regression results for NRs

Negative Relationships	*B*	95% CI for *B*		*SE B*	*R*^*2*^	*R*^*2*^_adjusted_	*p*
		*LL*	*UL*				
Model					0.39	0.35	< 0.001
Constant	8.54	3.06	14.01	2.70			0.003
Attachment avoidance	3.87	2.21	5.54	0.82			< 0.001
Attachment anxiety	− 2.09	− 3.77	− 0.40	0.83			0.017

Model = “Enter” method in SPSS statistics; *B* = unstandardized regression coefficient, CI = confidence interval; *LL* = lower limit, *UL* = upper limit, *SE B* = standard error of the coefficient; β = standardized coefficient; *R*^*2*^ = coefficient of determination; *R*^*2*^_adjusted_ = adjusted *R*^*2*^*; p* = probability value

### Qualitative analysis

Qualitative data was gathered to gain an understanding of factors participants deemed important to developing group cohesion. 71.05% (*n* = 27) of the participants responded to the question “What do you think leads to better group cohesion?” and 60.53% (*n* = 23) responded to the question, “Why is it important to you to experience good group cohesion?”. All responses were coded independently by two of the researchers, and from the coding, themes were identified. The five themes identified from the qualitative data are discussed below.

#### Safety, trust and vulnerability

Participants reported that a group environment where members feel safe to trust one another is important in promoting group cohesion. One participant stated that this helps *“people feel more comfortable to share freely without judgement”*. Participants reported that this allows members to be vulnerable and contribute in a genuine way in group discussions, and that this relationship is bi-directional. That is, safety, trust and vulnerability contribute to better group cohesion and vice versa. One participant stated, *“Let everyone talk and have a turn, have compassion for others, respect their story and journey, contribute as well”.* Behaviours such as all group members taking turns to contribute to discussion, as well as maintaining confidentiality by not sharing information outside of group were also considered beneficial to promoting group cohesion.

#### Compassion, support and respect

Participants reported that an atmosphere of compassion, support and kindness between members contributes to better group cohesion. They referred to the importance of an environment where members respect one another, and the group rules, as this fosters cohesion and facilitates fairness between members. One participant stated that if, *“Everyone respects the rules, take turns to share, arrive on time, no cross talking, [and] respect each other”*, this leads to better group cohesion. Members listening to other group members and the facilitator(s) whilst they are speaking was identified as important to promoting group cohesion. Participants highlighted that if group members support one another in their recovery goals, better cohesion is achieved, and similarly stronger cohesion leads to improved recovery outcomes for members. One participant stated, *“Support everyone in their goals, be kind, let others share, support new people”.*

#### Effective group facilitators

Many participants referred to the role that facilitators play in influencing group cohesion. One participant reported that better group cohesion is developed when, *“Facilitators involve everyone in the room”* and this was reflected by other participants. Another said that cohesion is promoted when, *“Facilitators are good at encouraging everyone to share and be non-judgmental”*. It was also reported that better group cohesion is developed if facilitators clearly explain things within group. Several participants referred to the importance of “good facilitators” in promoting group cohesion, however did not specify what constituted a “good facilitator”.

#### Belonging, acceptance and connection

Participants linked experiences of group cohesion to a sense of belonging, acceptance and connection between members. Several participants described not feeling understood by others outside of group and attending group allows them to “*have a space I feel understood”*. Another participant stated, *“If we aren't judged then we feel more belonging and people outside of group don't always really understand our struggles”*, highlighting that group cohesion allows the group to function as a space where members feel a sense of belonging and acceptance, which is not felt outside of group. Another participant described, “*If you don't belong elsewhere then you can here”.*

#### Shared goals

Several participants highlighted that shared goals and tasks in group promote group cohesion. In this particular group, the mutual goal of abstinence was identified as integral to better group cohesion by many members, along with shared home tasks and session goals that are set by facilitators. One participant stated that better cohesion is promoted if members, *“Have a goal for the session. Set homework so we work on the same thing”*.

## Discussion

The purpose of the current study was to explore the influence of attachment style on individuals’ experiences of group cohesion in an SUD therapy group, to determine whether attachment avoidance and attachment anxiety significantly predicted individuals’ ratings of PB, PW and NR. The current study also provided a qualitative exploration of factors that group members rated as important to promoting better group cohesion within SUD group therapy.

### Influence of attachment avoidance

The first key finding was that attachment avoidance, as hypothesised, was a significant negative predictor of PB and PW, and a positive predictor of NR. Participants who avoided intimacy and were distrusting of others were less likely to experience a sense of belonging or attraction to the group, its members and its leader(s), and reported lower constructive functioning on group tasks and goals. They were also more likely to experience a lack of trust, genuineness and understanding within group. This finding is consistent with existing research in non-SUD populations, where individuals stronger in attachment avoidance have reported lower group cohesion [26, 36]. These individuals hold negative internal working models of others, which lead them to perceive others as threatening and feel discomfort towards intimacy and emotional connection [41]. This likely impacts on the bonds that these members form with other members, their ability to work productively and collaboratively on tasks and group goals, and perhaps leads to a higher likelihood of negative interactions and relationships with other members in the therapy group.

### Influence of attachment anxiety

The second key finding was that attachment anxiety significantly predicted lower NR, however was not a significant predictor of PB and PW. As hypothesised, participants with a tendency to require excessive reassurance from others and fear rejection were less likely to report experiencing negative aspects of group relationships, such as group conflict, friction, distance and alliance ruptures. This is consistent with existing literature which posits that these individuals old a positive internal working model of others [41]. This may influence them to report positive group relationships, regardless of interpersonal conflict and friction, due to their strong desire to be seen favourably by others and a greater focus on others’ needs [31, 41, 55].

Unexpectedly, attachment anxiety did not significant predict higher PB or PW. As described earlier, there are mixed findings in the existing literature, with empirical support for both a positive and negative relationship between attachment anxiety and cohesion [8, 26, 36, 37]. The lack of relationship between attachment anxiety and both PB and PW in the current study may possibly be explained by methodological limitations. The current study design did not account for the aggregated influence of all members’ attachment styles, which has previously been found to play a role in how the individual experiences group cohesion [36, 38]. Further, the current study was unable to account for the individual stage of therapy of each participant due to the limited sample size, nor was it able to track changes in group cohesion over the course of the group. This may have impacted the findings, particularly as the number of previously attended group sessions of the participants ranged from 2 to 70 + sessions, indicating the likelihood of participants at various stages of recovery. Group cohesion has been found to fluctuate over the course of a group, and the trajectory has been found to differ between those stronger in attachment avoidance and those stronger in attachment anxiety [21, 24, 26, 58]. While it is possible that experiences of PB and PW for individuals with stronger attachment anxiety may be more sensitive to situational variables, such as other group members and length of time in group, it could be argued that likelihood of reporting lower NR may remain stable due to the individual’s internal working model of others as positive, which persists across time and situation. Similarly experiences of group cohesion for those with stronger attachment avoidance may be less affected by these situational variables, due to an internal negative working models of others, which means that perceptions and behaviours of others are less influential and important to these individuals. Future research would need to explore this further to elucidate whether this may explain the current findings.

### Qualitative exploration

The third key finding is the qualitative exploration of factors identified as promoting group cohesion in SUD therapy groups. The following themes were identified by participants as important to group cohesion, (a) safety, trust and vulnerability between members, (b) compassion, support and respect between members, (c) effective group facilitators, (d) belonging, acceptance and connection between members, and (e) shared group goals. The qualitative findings are consistent with existing literature, which proposes that the group may serve as a secure base that allows members to feel safe to be vulnerable and take emotional risks [41, 43] and that this may contribute to more effective individual and group functioning, which promotes healing and recovery. Further, the role of group facilitators in influencing group cohesion has been highlighted, as outlined in previous literature [26, 56]. The study also highlighted concrete behaviours that were identified by participants as beneficial to cohesion. These included all members contributing to group discussion, listening to other members and the facilitator(s) when they speak, respecting group rules, identifying mutual group tasks and goals, and the facilitator providing clear explanations during group. The findings also lend support to the quantitative results of the current study, highlighting that processes and behaviours that promote group cohesion, such as connecting with others, being vulnerable and developing trust towards the group, may be more difficult for individuals stronger in avoidant attachment. This may explain why they are less likely to experience group cohesion and the implications of this are described below.

### Limitations

Some methodological limitations of the current study have been outlined already. These should be considered when interpreting the current findings and future research should seek to address these limitations. As mentioned, analyses to determine whether client characteristics (e.g. demographic variables, number of previously attended sessions) and group characteristics (e.g. group size) influenced the relationship between attachment style and group cohesion were not possible due to the impact of COVID-19 on participant recruitment. Variables such as age, gender, race and group size have been found to influence group behaviours, as well as the relationship between group cohesion and treatment outcomes, thus future research should seek to incorporate this in their study design to clarify their role in SUD group therapy [13, 16, 57]. The current sample also included participants with high income and education, who attended an open-group program. This may impact on generalisability of findings to other populations with lower income and education, or those who attend closed-group programs where the trajectory of group cohesion may differ. Further research should seek to account for this to contribute to a more robust understanding of the influence of attachment style on group cohesion in the literature. The current study was also unable to explore the influence of healthy, secure attachment on experiences of group cohesion and future research should seek to further address this to provide a better understanding of how securely attached individuals experience group SUD treatment.

### Implications and directions for future research

Research exploring the impact of attachment style on group cohesion in SUD group therapy is important to improving therapeutic outcomes. The current findings have several practical implications for group therapists providing SUD treatment. The present findings highlight the importance of therapists tailoring group treatment to meet the attachment needs of individuals to minimise barriers to successful therapeutic engagement and positive treatment outcomes. The study also provides qualitative information on processes and behaviours that individuals with lived experience of SUD have identified as beneficial to group cohesion, which therapists can take into consideration. As individuals stronger in attachment avoidance may have greater difficulty engaging in behaviours that promote group cohesion, future research should explore how therapists can account for avoidant attachment styles in group therapy, and particular ways that they can support these members to experience the group as a safe base. Future research should also seek to clarify the relationship between attachment anxiety and group cohesion given the mixed findings in the existing literature, accounting for the methodological limitations of the current study, as well as the broader methodological challenges of group cohesion research. The current study, along with future research in this area, will allow group therapists to enhance their capacity to provide effective, individualised treatment for SUD in the group format.

## Data Availability

The data and code utilised in this study is available on request from the corresponding author. The data is not publicly available due to their containing information that could compromise the privacy of research participants.
